# Combined action observation and mental imagery versus neuromuscular electrical stimulation as novel therapeutics during short‐term knee immobilization

**DOI:** 10.1113/EP091827

**Published:** 2024-04-30

**Authors:** Kylie K. Harmon, Ryan M. Girts, Gabriela Rodriguez, Jonathan P. Beausejour, Jason I. Pagan, Joshua C. Carr, Jeanette Garcia, Michael D. Roberts, Debbie L. Hahs‐Vaughn, Jeffrey R. Stout, David H. Fukuda, Matt S. Stock

**Affiliations:** ^1^ Department of Exercise Science Syracuse University Syracuse New York USA; ^2^ Department of Natural and Health Sciences Pfeiffer University Misenheimer North Carolina USA; ^3^ Institute of Exercise Physiology and Rehabilitation Science, School of Kinesiology and Rehabilitation Sciences University of Central Florida Orlando Florida USA; ^4^ Department of Kinesiology Texas Christian University Fort Worth Texas USA; ^5^ Department of Medical Education Anne Burnett Marion School of Medicine at Texas Christian University Fort Worth Texas USA; ^6^ School of Sport Sciences West Virginia University Morgantown West Virginia USA; ^7^ School of Kinesiology Auburn University Auburn Alabama USA; ^8^ Department of Learning Sciences and Educational Research University of Central Florida Orlando Florida USA

**Keywords:** corticospinal, disuse atrophy, muscle mass, nervous system, rehabilitation, strength

## Abstract

Limb immobilization causes rapid declines in muscle strength and mass. Given the role of the nervous system in immobilization‐induced weakness, targeted interventions may be able to preserve muscle strength, but not mass, and vice versa. The purpose of this study was to assess the effects of two distinct interventions during 1 week of knee joint immobilization on muscle strength (isometric and concentric isokinetic peak torque), mass (bioimpedance spectroscopy and ultrasonography), and neuromuscular function (transcranial magnetic stimulation and interpolated twitch technique). Thirty‐nine healthy, college‐aged adults (21 males, 18 females) were randomized into one of four groups: immobilization only (*n* = 9), immobilization + action observation/mental imagery (AOMI) (*n* = 10), immobilization + neuromuscular electrical stimulation (NMES) (*n* = 12), or control group (*n* = 8). The AOMI group performed daily video observation and mental imagery of knee extensions. The NMES group performed twice daily stimulation of the quadriceps femoris. Based on observed effect sizes, it appears that AOMI shows promise as a means of preserving voluntary strength, which may be modulated by neural adaptations. Strength increased from PRE to POST in the AOMI group, with +7.2% (Cohen's *d* = 1.018) increase in concentric isokinetic peak torque at 30°/s. However, NMES did not preserve muscle mass. Though preliminary, our findings highlight the specific nature of clinical interventions and suggest that muscle strength can be independently targeted during rehabilitation. This study was prospectively registered: ClinicalTrials.gov NCT05072652.

## Abstract (highlights)

- **What is the central question of this study?**
  Action observation and mental imagery (AOMI) may be a possible and potentially promising rehabilitation strategy via facilitation of neural adaptations, whereas neuromuscular electrical stimulation (NMES) may augment muscle strength and mass via peripheral mechanisms. We compared the ability of these interventions to distinctly preserve strength versus mass during one week of knee joint immobilization.
- **What is the main finding and its importance?**
  Effect sizes suggested that AOMI preserved voluntary strength, and may be used therapeutically to target neural adaptations during immobilization.

## 1 INTRODUCTION

Periods of immobilization and disuse result in rapid declines in muscle strength and mass (Clark, 2009; Deitrick, 1948; Gibson et al., 1987; Ingemann‐Hansen & Halkjaer‐Kristensen, 1980; Wall et al., 2014). Knee joint immobilization is a commonly used clinical intervention for recovery after orthopedic surgery (e.g., total knee arthroplasty) or injury. While immobilization plays a crucial role in the healing process by protecting the injured limb and allowing time to heal, it is not without consequences. Recovery from periods of immobilization can be challenging, as the time required to return to baseline strength levels can be extensive, which may impair activities of daily living and temporarily impact quality of life. In an effort to mitigate these deleterious effects, much attention has been given to the physiological changes that occur throughout periods of immobilization and disuse. While both muscle strength and mass are negatively impacted by disuse, research suggests that loss of strength occurs more rapidly than loss of muscle mass (Dirks et al., 2014; S. W. Jones et al., 2004; Thom et al., 2001; White et al., 1984). Hence, attempts to preserve muscle mass and strength during periods of immobilization likely require distinct or combined interventions.

Given the discrepancy between muscle mass and strength loss, it is likely that neuromuscular maladaptation is at least partially responsible for these observations. It has long been recognized that neural adaptations play a critical role in the initial stages of strength gains (Moritani & deVries, 1979), such as improved motor unit firing patterns, enhanced voluntary activation (VA), decreased coactivation of antagonist muscles, and increased corticospinal excitability and decreased inhibition (Gabriel et al., 2006; Kidgell et al., 2017). Further, it has more recently been suggested that neuromuscular deconditioning is responsible for the early rapid changes in strength observed during disuse (Campbell et al., 2019). Consequently, in an effort to preserve strength during immobilization, several studies have investigated the neuromuscular mechanisms responsible for strength loss (Clark et al., 2014; Cook et al., 2014), rather than muscle atrophy alone. Two neurophysiological techniques, those of action observation (AO) and mental imagery (MI), have demonstrated promise. Both AO and MI are neural intervention strategies wherein a participant observes or imagines an action, with no actual muscular contraction occurring. Rather than targeting the muscle tissue or causing muscular contraction, these strategies target the nervous system and elicit similar activity in the motor system without actual motor execution (Eaves et al., 2016). Although there is significant overlap between active neural structures during both AO and MI, subtle differences do exist. The action observation network in particular includes structures involved in the mirror neuron system (Mizuguchi & Kanosue, 2017; Rizzolatti & Craighero, 2004). Mirror neurons are specialized types of neurons found in the human frontal, parietal and temporo‐occipital cortices (Caspers et al., 2010) that fire not only when an individual performs an action, but when they observe actions performed by another (di Pellegrino et al., 1992; Rizzolatti & Craighero, 2004; Rizzolatti et al., 1996). Multiple studies utilizing transcranial magnetic stimulation (TMS) to probe the motor cortex have observed increased corticospinal excitability in response to AO interventions (see Loporto et al., 2011; Naish et al., 2014 for review).

Of particular interest is how these two forms of motor simulation activate the motor system, and the motor cortex in particular (Hétu et al., 2013). The motor cortex has only recently been recognized for its role in strength production, yet the findings are encouraging. Several studies have demonstrated that MI training increases strength in the absence of contraction (Fontani et al., 2007; Ranganathan et al., 2004; Yue & Cole, 1992; Zijdewind et al., 2003). Further, MI training may attenuate unfavourable corticospinal and strength changes that occur with disuse (Clark et al., 2014, 2010). Throughout a 4‐week hand and wrist immobilization protocol, Clark et al. (2014) observed that regular MI of strong muscle contractions throughout immobilization attenuated the loss of strength and VA by ∼50% compared to those who underwent immobilization without MI intervention. In those that did not perform MI training, muscle weakness was strongly associated with decreased VA and increased corticospinal inhibition.

AO interventions have also resulted in improved functional outcomes and motor performance. Like MI, AO requires no muscular contraction, as a participant simply observes another's actions. AO has been shown to improve functional outcomes when utilized during motor rehabilitation (Bellelli et al., 2010; Marusic et al., 2018) and enhance strength during MI training (Scott et al., 2018). Interventions utilizing TMS have demonstrated increased corticospinal excitability when AO and MI are used in conjunction (Sakamoto et al., 2009; Wright et al., 2014). Thus, throughout periods of immobilization, the combination of AO and MI (AOMI) may be more effective at preserving strength via neuromuscular mechanisms than either intervention alone.

Interventions that attempt to preserve muscle mass throughout periods of immobilization often focus on changes at the tissue level. One such intervention is that of neuromuscular electrical stimulation (NMES). NMES invokes involuntary muscle contractions, allowing for muscle activity without joint movement. In addition to its ability to act as a surrogate for voluntary muscle contraction, NMES increases muscle protein synthesis (Wall et al., 2012). Given this, NMES has gained traction in the literature as a method of potentially preserving muscle mass during periods of immobilization (Dirks et al., 2014; Slysz et al., 2021), disease (Dirks et al., 2015; S. Jones et al., 2016) or recovery from injury (Spector et al., 2016). However, despite preservation of muscle mass and recent evidence of positive effects on the central nervous system (Maffiuletti et al., 2018), NMES does not appear to mitigate strength loss during short‐term limb immobilization (Dirks et al., 2014; Slysz et al., 2021).

Given the physical deficits, recovery time, and health care burden (CDC–NCHS–National Center for Health Statistics, 2024) of disuse‐induced deficits, interventions that mitigate muscle weakness and atrophy are needed. AO, MI and NMES are accessible and affordable interventions, and their use could be widely implemented in clinical settings or even at home in the absence of direct supervision. While the aforementioned findings are promising, no study has yet directly compared the use of AOMI and NMES throughout an immobilization protocol. If previous findings are replicated, preservation of muscle strength versus mass throughout immobilization could decrease rehabilitation time and improve recovery outcomes. Therefore, the purpose of this study was to compare the effects of AOMI versus NMES on muscle strength, muscle mass and neuromuscular function during 7 days of knee joint immobilization. We hypothesized that AOMI would have a preservatory effect on strength via maintenance of neuromuscular function, while NMES would have a preservatory effect on muscle mass.

## 2 METHODS

### 2.1 Study design

All study protocols described herein were approved by the University Institutional Review Board prior to participant enrollment (STUDY00003289) and conformed to the standards set by the latest revisions of the *Declaration of Helsinki*. This study was prospectively registered on ClincalTrials.gov (Identifier: NCT05072652). This investigation utilized a between‐participants design in which muscle strength and mass of the left (immobilized) lower limb was assessed before and after 1 week of knee joint immobilization. The left limb was chosen as the immobilized limb to allow participants to operate a motor vehicle as needed. Before data collection, participants underwent a thorough familiarization (FAM) visit to ensure comprehension of study protocols and minimize the influence of a learning effect. Data were collected during baseline testing (PRE) and 1 week later (POST). All visits to the laboratory occurred at the same time of day (±1 h). All image capture and analyses, as well as all TMS assessments, were performed by the same investigator. Throughout the study, participants were asked to refrain from alcohol, keep their dietary habits consistent, and comply with the immobilization protocols and assigned intervention. To ensure compliance and safety, each participant was paired with a member of the research team, who served as their compliance officer. Each compliance officer checked‐in with their assigned participants daily and assisted with any concerns or issues that arose. Laboratory tests (described below in the order in which they were performed) included bioimpedance spectroscopy (BIS), ultrasonography, isometric maximal voluntary contractions (MVCs), assessment of VA, assessment of motor cortex hotspot and active motor threshold (AMT) via TMS, and concentric isokinetic strength assessments.

After study enrollment, a random number generator was used to assign participants to one of four groups in a randomized design: immobilization only, immobilization + AOMI, immobilization + NMES, or a control group that did not undergo immobilization. Each group was stratified according to sex, with the goal of an even distribution of males and females assigned to each group. Both the investigators and the participants were blinded to group assignment during the screening process. Group assignment for each participant was determined during their first visit to the lab.

### 2.2 Participants

**FIGURE 1:**
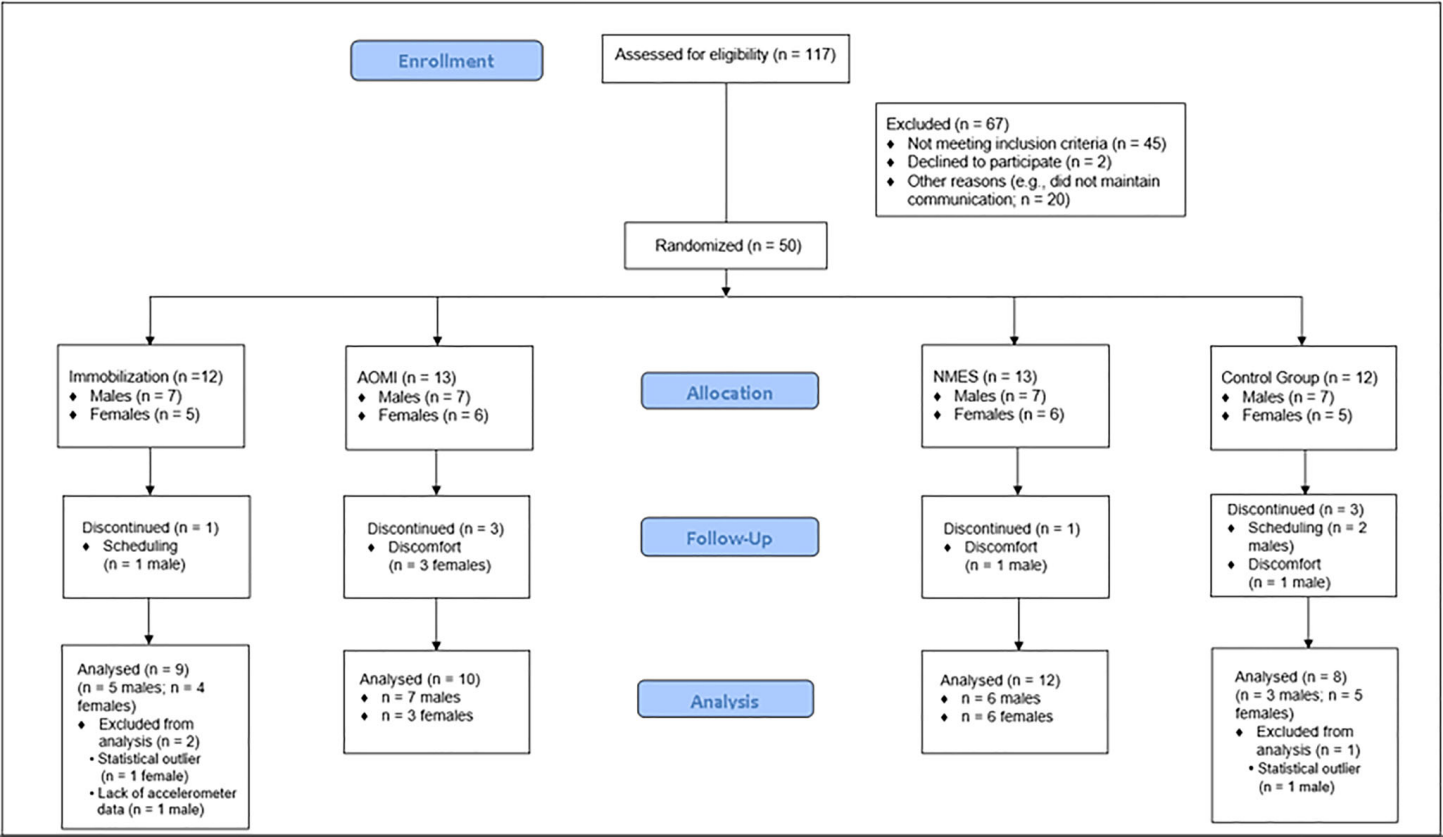
Flow diagram of individuals screened, enrolled and included in the final analyses.

A sample of 50 healthy adults enrolled in this study. Of those that enrolled, eight participants elected to not return after the FAM visit, due to scheduling conflicts (*n* = 3) or discomfort with the study procedures (*n* = 5). Of the 42 participants who completed all study procedures, one was removed due to an accelerometer issue which prevented confirmation of compliance (i.e., only one accelerometer was recording data throughout the immobilization period). Two participants were removed as statistical outliers based on percentage change of MVC peak torque from PRE to POST testing. Further detail on the statistical procedures for outlier removal can be found in the statistical analysis section. The final participant sample can be found in Figure 1.

Thirty‐nine healthy adults (21 males, mean ± SD age = 22 ± 3 years; height = 180.26 ± 7.04 cm; mass = 80.07 ± 13.28 kg; body mass index (BMI) = 24.61 ± 3.15 kg/m^2^; 18 females, mean ± SD age = 20 ± 1 years; height = 164.19 ± 8.04 cm; mass = 64.54 ± 10.72 kg; BMI = 23.83 ± 2.32 kg/m^2^) completed all study procedures and were included in final data analyses.

Prior to study enrollment, participants were required to complete a detailed health history questionnaire and a TMS‐specific screening questionnaire to ensure no contraindications to participation existed. Exclusion criteria included BMI below 18.5 or above 35.0 kg/m^2^, musculoskeletal issues (i.e., back, shoulder or knee pain) that may have interfered with the use of crutches, recent history of surgery on the hip or knee joints, family history of thrombosis, history of seizures or fainting, use of certain medications (i.e., muscle relaxants or benzodiazepines), use of a cardiac pacemaker, or current pregnancy. Given previous investigations indicating that training status does not impact the magnitude of disuse‐induced deficits (Deschenes et al., 2017; Deschenes, McCoy, Davis, et al., 2009), both trained and untrained participants were enrolled. All participants were notified of the study risks and completed an informed consent document prior to enrollment. Depending on group assignment, participants were compensated up to US $120 for their time, at a rate of US $10–20 for FAM and PRE visits and US $10–90 for POST. For completing all study procedures, the control group received US $30, the immobilization‐only group received US $100, and the AOMI and NMES groups received US $120. It should be noted that this was Phase 1 of a two‐phase study. Participants received additional compensation for Phase 2, which could total US $220 for completion of the entire study, as well as a T‐shirt (Girts et al., 2024).

### 2.3 Anthropometrics and body composition

During the first laboratory visit, body mass and height were assessed using a physician's scale and stadiometer (Seca 700, Chino, CA, USA). During each subsequent visit (PRE and POST), body mass was obtained at the start of each visit for use in BIS analyses. Segmental lean mass of the left leg was assessed by BIS using the ImpediMed SFB7 (ImpediMed Inc., Carlsbad, CA, USA). Participants rested in the supine position for at least 5 min prior to testing while electrodes were placed according to the manufacturer's recommendations. Briefly, electrodes were placed on the left foot and thigh, at the level of the malleolus and 5 cm distal from the greater trochanter, respectively. Additional electrodes were placed 5 cm distal to each landmark. Height, mass and sex information was entered into the device prior to each test. Measurements were taken twice while participants lay supine with their arms ≥30 degrees away from their torso and their legs separated. Segmental lean mass was determined according to the following equation: segmental lean mass (kg) = 4.44 + 1.21 × ICV (L), where ICV is intracellular volume. For further detail, the reader is directed to the work of Kaysen et al. (2005).

### 2.4 Ultrasonography measurements and analyses

B‐mode ultrasonography images of the vastus lateralis (VL) and rectus femoris (RF) muscles of both limbs were taken with a portable imaging device (GE Logiq BT12, GE Healthcare, Milwaukee, WI, USA) and a multi‐frequency linear‐array probe (12 L‐RS, 5–13 MHz, 38.4‐mm field of view; GE Healthcare, Milwaukee, WI, USA). Prior to image capture, participants rested supine for 5 min to allow the redistribution of fluids from their quadriceps muscles (Arroyo et al., 2018). The panoramic function (LogiqView, GE Healthcare, Milwaukee, WI, USA) was used to obtain images of the VL and RF in the transverse plane. Measurements for the RF were taken at 50% of the distance from the anterior, inferior suprailliac spine to the most proximal point of the patella. VL measurements were taken at 50% of the straight‐line distance between the greater trochanter and the lateral epicondyle of the femur. A high‐density foam pad was secured around the thigh with an adjustable strap to ensure desired probe movement in the transverse plane. Ultrasonography settings (frequency: 12 MHz, gain: 55 dB, dynamic range: 72) were kept consistent across participants. To ensure optimal image clarity, a standardized depth of 5.0 cm was utilized unless a greater depth was necessary to adequately capture the entire belly of the muscle (Girts et al., 2022). Depth for each participant was kept consistent across trials. A generous amount of water‐soluble transmission gel (Aquasonic 100 ultrasonography transmission gel, Parker Laboratories, Inc., Fairfield, NJ, USA) was applied to the skin to allow immersion of the probe surface during measurement to enhance acoustic coupling. Three images of each muscle were obtained, with mean values being used for statistical analyses.

The ultrasonography images were digitized and examined with ImageJ software (version 1.46, National Institutes of Health, Bethesda, MD, USA) at the conclusion of the study. The polygon function was used to outline the borders of transverse images of the VL and RF. After scaling the image units from pixels to cm, muscle cross‐sectional area (CSA) (cm^2^) was determined with the polygon function. The same experienced researcher obtained and analysed all ultrasound images (Carr et al., 2021), and demonstrated excellent intrarater reliability (intraclass correlation coefficient (ICC) = 0.988, SEM = 0.68%).

### 2.5 Assessment of isometric torque

All isometric strength measurements were performed with the left (immobilized) knee extensors via a Biodex System 4 isokinetic dynamometer (Biodex Medical Systems, Shirley, NY, USA). Before testing, participants were seated in the dynamometer with restraining straps placed over the trunk, pelvis and thigh. The input axis of the dynamometer was aligned with the axis of rotation of the knee. Each participant's dynamometer chair settings were recorded and replicated during subsequent testing sessions. The chair was adjusted so that isometric torque testing was performed at hip and knee joint angles of 90°. The lower leg was secured to an anti‐shear attachment with the pad placed over the tibialis anterior, just superior to the malleoli. Prior to testing, participants were given a warm‐up consisting of three 10‐s contractions at 50%, one 5‐s contraction at 75%, and one 3‐s contraction at 90% of their perceived maximal torque.

After the warm‐up, participants performed one 5‐s MVC of the knee extensors. Participants were instructed to push as hard and fast as possible. During the MVC, participants received visual feedback of their torque output on a screen placed at eye‐level ∼1.5 m in front of them, as well as strong verbal encouragement from the research team. The highest recorded value was designated as the MVC peak torque value (N∙m) according to Delsys EMGWorks software (version 4.7.5, Delsys, Inc., Natick, MA, USA).

### 2.6 Voluntary activation

VA was determined using electrical stimulation of the knee extensor muscles during MVCs, based on the methodology of MacLennan et al. (2021). Stimulation was delivered with adhesive surface electrodes placed over the VL and vastus medialis, about one‐third and two‐thirds the distance from the greater trochanter to the superior aspect of the patella (MacLennan et al., 2021; Park et al., 2008; Pietrosimone et al., 2011). For testing, the electrodes were connected to a constant‐current stimulator (DS7AH Digitimer, Welwyn Garden City, UK). While at rest, the optimal electrical current needed to elicit maximal involuntary torque from the quadriceps femoris muscles was determined by a series of increasing electrically stimulated contractions. Paired pulse stimulation was used, with two 200 μs pulses separated by 10 ms. The first stimulation was set at 100 mA, with each successive stimulation increased by 20 mA. There was at least 10 s between successive stimulations. Optimal stimulation intensity was established when the elicited peak torque resulted in two consecutive decreases and was determined at each testing session.

The interpolated twitch technique (ITT) was used during three additional MVCs (MacLennan et al., 2021). When torque plateaued, a stimulation at the previously identified intensity was delivered, and the increase in involuntary torque was measured. Upon feeling the stimulation, participants were instructed to relax, at which point two additional stimulations were delivered at approximately 2 and 4 s after the MVC. The mean of these two values was used to establish electrically evoked torque (EET). VA was calculated as [1 − (ITT/EET)] × 100. The mean of the VA trials was used for subsequent analyses.

### 2.7 Surface electromyography

Throughout testing, bipolar surface electromyography (EMG) sensors (Trigno EMG, Delsys) were placed over the belly of the left VL. Specific sensor placement was based on the recommendations described in the Surface EMG for the Non‐Invasive Assessment of Muscles (SENIAM) project (Hermens et al., 2000). Prior to placement of each sensor, the skin was shaved with a disposable razor, cleared of any oil or debris with hypo‐allergenic tape, and cleansed with isopropyl alcohol. EMG sensors were attached to the surface of the skin with adhesive tape. Prior to testing, participants performed several submaximal contractions to ensure low baseline noise (≤20 μV peak‐to‐peak amplitude) and minimal line interference. EMG signal quality was monitored throughout the study, with additional skin preparation or repositioning of sensors performed, as necessary.

### 2.8 M‐wave determination

Throughout VA testing, surface EMG signals from the VL were monitored in response to increasing electrical stimulation. The EMG signal with the highest peak‐to‐peak amplitude was recorded as the maximum compound muscle action potential (M‐wave) value (μV). This value was used for normalization of TMS‐induced motor evoked potentials (MEPs) as described below.

### 2.9 Transcranial magnetic stimulation

Single‐pulse TMS was performed using a MagStim 200^2^ (The MagStim Co., Whitland, UK) stimulator and a double‐cone coil. TMS pulses were delivered while participants performed submaximal contractions of the left knee extensors (Kesar et al., 2018). Participants performed submaximal isometric contractions at 10% of their previously determined MVC peak torque by tracing a trajectory on the screen in front of them. They maintained this submaximal effort throughout all TMS assessments. To avoid the accumulation of fatigue, breaks were given as needed.

The hotspot was determined as the location over the right hemisphere of the motor cortex that elicited the largest peak‐to‐peak amplitude for the left (immobilized) VL MEPs. Once identified, the hotspot location was marked on a Lycra cap to ensure consistent coil placement. After determining the hotspot, AMT was determined as the lowest stimulator intensity that could reliably produce a MEP of at least twice the baseline EMG signal for 5 out of 10 TMS pulses (Clark et al., 2008; Harmon et al., 2022). Twenty single TMS pulses were delivered to the hotspot at a stimulator intensity corresponding to 130% AMT (Clark et al., 2008; Damron et al., 2008; Goss et al., 2012). Hotspot and AMT were determined at both PRE and POST visits.

MEP amplitude and silent period duration were then used to quantify corticospinal excitability and inhibition, respectively. The peak‐to‐peak amplitude of the MEPs was determined, and normalized to VL M‐wave by dividing the MEP value (μV) by the M‐wave value (μV), with the final value multiplied by 100 and expressed as a percentage. Silent period duration was quantified as the amount of time between the delivery of the TMS pulse and return of surface EMG signal. This was done manually with custom LabVIEW software (version 20.0, National Instruments, Austin, TX, USA). The same investigator performed all MEP amplitude and silent period duration analyses and demonstrated excellent intrarater reliability (ICC = 0.998, SEM = 0.48%).

### 2.10 Assessment of concentric isokinetic torque

Following the TMS procedures, participants performed dynamic strength tests of the left knee extensors on the Biodex isokinetic dynamometer. Specifically, five maximal concentric isokinetic muscle actions at 30°/s and 180°/s were performed with at least 2 min of rest between each velocity. Participants were encouraged to push ‘hard and fast’ throughout the full range of motion of concentric knee extension. Custom LabVIEW software (version 20.0, National Instruments, Austin, TX) was used to visually determine the repetition with the highest peak torque, which was used for further analysis.

### 2.11 Signal processing

All torque, TMS, and EMG signals were acquired in‐sync with Delsys EMGWorks Software (version 4.7.5). Surface EMG sensors had a bandwidth of 20–450 Hz, an input range of 11 mV, and sampling rate of 1926 Hz. Concentric isokinetic peak torque and EMG signals were processed off‐line using custom LabVIEW software (version 20.0).

### 2.12 Immobilization procedures

At the end of PRE, participants assigned to immobilization groups were fitted with a knee joint immobilization brace (T Scope Premier Post‐Op Knee Brace, Breg, Inc., Carlsbad, CA, USA). The brace was locked at 90° of knee flexion, to ensure that the foot was raised off the ground. This position prevented normal weight‐bearing and allowed the knee extensors to stay relaxed. Participants were instructed to only remove the brace in bed, prior to sleep. During bathing, participants were instructed not to remove the brace, but to keep it dry by covering it with a large plastic bag provided by the research team. For their safety and comfort, each participant was offered a shower chair (Medline Shower Chair Bath Seat with Padded Armrests and Back, Medline Industries, Inc., Northfield, IL, USA) to assist in bathing. For ambulation, participants were provided with axillary crutches (Cardinal Health Axillary Crutch, Adult, Adjustable, Cardinal Health, Inc., Dublin, OH, USA). Participants were fitted with and trained in the proper use of crutches, including navigation of stairs, doors and other community obstacles.

Participants were given a stocking (McKesson Tubular Cotton Stockinettes, San Francisco, CA, USA) to wear underneath the brace, which was measured to extend from the proximal thigh to the ankle. The stocking was intended to mitigate discomfort and minimize the risk of skin irritation from the brace. This was worn at all times and only removed during sleep when the brace was removed. A compression stocking (Medi‐Pak Anti‐Embolism Stockings) was provided to be worn while sleeping, to reduce the risk of blood clots.

In accordance with previous knee joint immobilization studies (Deschenes et al., 2008; Deschenes, McCoy, Holdren, et al., 2009; MacLennan et al., 2021, 2020), participants performed twice daily (morning and evening) range of motion movements of the ankle and knee to minimize the risk of vascular or muscular issues due to immobilization. Movements were performed while lying in bed, and consisted of knee flexion, ankle pumps (dorsiflexion and plantarflexion) and leg lowers. A video providing instructions was provided to participants.

### 2.13 Action observation/mental imagery

Participants in the AOMI group performed one daily session of AO followed immediately by MI. Participants received a link to a private channel on an online video sharing platform and were instructed to watch this video and follow the on‐screen instructions daily. During both the AO and MI interventions, participants were instructed to maximally activate the brain to imagine that they were flexing the muscles in their left leg as hard as possible, without actually contracting the muscle, and to refrain from performing the actions (Clark et al., 2014; Yue & Cole, 1992).

**FIGURE 2:**
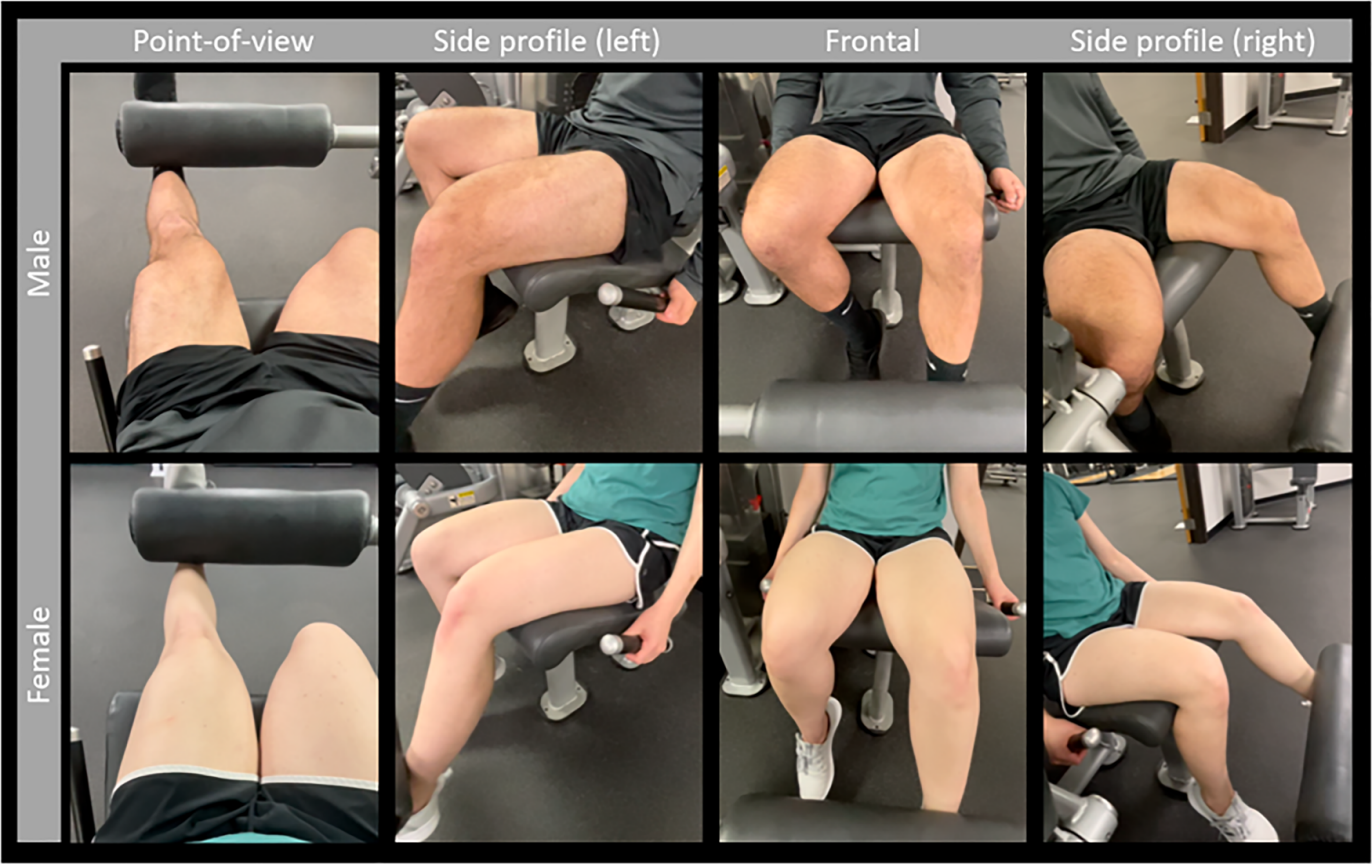
Representation of perspectives included in the AO video.

During the AO video observation, participants were instructed to observe the on‐screen actions and imagine that they were performing the movements themselves. Participants observed videos of a sex‐matched adult performing heavy leg extension exercises from a variety of perspectives: point of view, side profile (left), frontal and side profile (right), as seen in Figure 2. The AO videos included multiple views/perspectives to best recruit all view‐dependent mirror neurons (Buccino, 2014; Caggiano et al., 2011). The face of the actor was not visible. Participants were instructed to use kinaesthetic imagery (i.e., imagining the physical sensations associated with performing the on‐screen movements) as this method of AO has been demonstrated to modulate corticospinal excitability to a greater extent than visual imagery alone (Stinear et al., 2006; Wright et al., 2014). Each AO session was ∼20 min long.

Immediately following the AO session, participants were instructed to begin MI. MI sessions were based on the work of Clark et al. (2014), in which an MI training protocol significantly attenuated reductions in strength and VA during immobilization (Clark et al., 2014). For each MI session, participants were instructed to perform 50 imagined contractions of the knee extensors. The duration of each imagined contraction was 5 s, followed by 5 s of rest. MI training was performed in two blocks of 25 contractions each, with 2 min rest between blocks. Total daily time for MI training was ∼10 min.

### 2.14 Neuromuscular electrical stimulation

For participants assigned to the NMES group, two NMES sessions were performed each day for the duration of the immobilization period. Participants did not report to the laboratory for NMES, but performed the sessions on their own, after being thoroughly familiarized with the NMES protocol during FAM, with additional review at the end of PRE. This protocol was based on the work of Dirks et al. (2014), during which twice daily NMES had a preservatory effect on muscle mass during 5 days of knee joint immobilization. NMES sessions were performed in the morning (07.00–12.00 h) and the afternoon (13.00–18.00 h), with a minimum of 4 h between sessions. Electrodes were placed on the distal and proximal ends of the muscle belly of the RF and VL and traced with a permanent marker before participants left the laboratory at PRE. Participants received a permanent marker to remark electrode position as needed, to ensure that the location of the electrodes was not altered between sessions.

Stimulation was provided by a commercially available NMES device (Omega Professional Tens and NMES/EMS Combo Unit, The Original Tens Units, Largo, FL, USA) and four self‐adhesive electrodes. The NMES protocol consisted of a warm‐up phase (5 min, 5 Hz, 250 μs), a stimulation period (30 min, 100 Hz, 400 μs) of 5 s on (0.75 s rise, 3.5 s contraction, 0.75 s fall) and 10 s off, and a cool‐down phase (5 min, 5 Hz, 250 μs). Participants were instructed to set the intensity of the stimulation to a level at which full contractions of the quadriceps femoris were visible. Additionally, participants were encouraged to increase the intensity of the stimulation during subsequent sessions to provide a progressive stimulus (Dirks et al., 2014).

### 2.15 Measurement of compliance

To ensure compliance with the immobilization protocol, participants were fitted with an Actigraph GTX9 accelerometer (ActiGraph Inc., Pensacola, FL, USA) on both ankles. Participants were instructed to wear the accelerometers for the entire duration of the immobilization week, to be removed only when showering or bathing. Accelerometer data monitored overall wear‐time compliance utilizing criteria established by Troiano (2007), and compliance with the immobilization protocol by examining differences in physical activity intensity and step counts between the right and left (immobilized) leg (Cook et al., 2006).

### 2.16 Nutritional intake

Throughout the immobilization week, participants completed a food and hydration log that interrogated two weekdays and one weekend day of intake. Participants were shown a ‘good’ and ‘bad’ example of completing the logs, and researchers instructed them to be as detailed as possible, by including brand names, amounts and preparation method of the food. Participants were also instructed that they could send a picture of the food to the researchers or compliance officers if they needed help logging the food accurately. When food log entries were vague (e.g., failed to specify brand or serving size), well known national brands were prioritized, and a standard single serving was used. After food logs were collected, the same member of the research team utilized a nutrition tracking app (My Fitness Pal, My Fitness Pal, Inc., version 22.7.5.39110) to sum daily calorie and protein intake, and the mean calorie and protein content of the three days was used for analysis.

### 2.17 Control group

The control group was instructed to adhere to the same restrictions and requirements as the experimental groups, apart from immobilization and the associated procedures. Like the experimental groups, participants in the control group were instructed to complete a food and hydration log, avoid alcohol and exercise, and keep dietary habits consistent for the duration of the study. However, unlike those in the experimental groups, participants in the control group were contacted by their compliance officers every other day, rather than daily. This was to ensure that no major changes occurred and that participants were complying with all study protocols.

### 2.18 Statistical analyses

The dependent variables of interest in this study included MVC peak torque (N∙m), VA (%), EET (N∙m), concentric isokinetic peak torque at 30°/s and 180°/s (N∙m), AMT (percentage of stimulator output), VL MEP peak‐to‐peak amplitude (μV) normalized to VL M‐wave (%), VL silent period duration (ms), segmental lean mass (kg), and VL and RF CSA (cm^2^). Prior to further statistical analysis, the presence of outliers was detected via the median absolute deviation method (Leys et al., 2013). This method was chosen due to the large influence of outliers on mean and standard deviation (SD). We elected to base outlier removal on percentage change in MVC peak torque from PRE to POST, as MVC peak torque was the primary outcome variable to detect changes in strength. Briefly, observations were sorted in ascending order and the median value was determined. The median was then subtracted from each observation and absolute values were determined. The median was then multiplied by a predetermined constant (1.4826) to determine the median absolute deviation (Leys et al., 2013). We then defined our rejection criteria as very conservative (i.e., 3), and multiplied the median absolute deviation by this criterion value. This value was then added to or subtracted from the median. Any values above or below the resulting value were removed as statistical outliers.

The variables of interest were analysed via multiple analysis of covariance (ANCOVA) tests to determine differences between the groups due to the immobilization interventions. Before further analyses, regression analysis was performed to ensure all assumptions associated with ANCOVA, such as such as random and independent residual errors, homogeneity of variance, normality, linearity of *x* and *y*, homogeneity of regression slopes, and independence of covariate and independent variable, were not violated. Pre‐test data served as the covariate in the analyses. Post‐test data served as the dependent variable and group served as the independent variable. Within‐group changes were determined with Student's *t*‐test for paired samples. To examine nutritional intake, a one‐way analysis of variance (ANOVA) was used to determine differences between groups in total calorie and protein intake.

When appropriate, follow‐up analyses included Bonferroni *post hoc* comparisons. An α‐level of 0.05 was used to determine statistical significance for all ANCOVAs and Bonferroni *post hoc* comparisons. The partial eta squared statistic (η_p_^2^) was used as a measure of effect size for each ANCOVA, with values of 0.01, 0.06 and 0.14 representing small, medium and large effects, respectively (Cohen, 1988). Cohen's *d* effect sizes were used to highlight important pairwise differences, with values of 0.2, 0.5 and 0.8 corresponding to small, medium and large effects, respectively (Cohen, 1988). All statistical procedures were performed with JASP software (version 0.14.1, University of Amsterdam, Amsterdam, The Netherlands).

## 3 RESULTS

All assumptions associated with ANOVA and ANCOVA, such as random and independent residual errors, homogeneity of variance, normality, linearity of *x* and *y*, homogeneity of regression slopes, and independence of covariate and independent variable, were not violated, and the analyses proceeded. Between‐group differences are reported in text below, with PRE to POST values found in Table 1. Within group differences are reported in Table 2.

**TABLE 1:** PRE to POST values (means ± SD).

	Immobilization		AOMI		NMES		Control	
	PRE	POST	PRE	POST	PRE	POST	PRE	POST
MVC Peak torque (N∙m)	183.6 ± 60.2	160.2 ± 56.3	165.5 ± 44.8	150.9 ± 38.4	154.7 ± 53.7	142.4 ± 54.2	176.5 ± 39.2	167.6 ± 38.9
VA (%)	97.1 ± 3.4	93.2 ± 4.5	88.5 ± 8.5	84.6 ± 14.3	92.8 ± 7.4	94.0 ± 6.4	92.9 ± 7.0	95.5 ± 5.0
EET (N∙m)	79.1 ± 26.1	79.4 ± 28.7	82.1 ± 24.5	81.2 ± 29.2	57.5 ± 16.3	62.8 ± 19.8	69.8 ± 23.0	67.4 ± 21.8
Peak torque 30°/s (N∙m)	149.8 ± 36.4	142.3 ± 35.3	120.3 ± 36.8	129.0 ± 39.8	126.8 ± 40.4	125.1 ± 39.7	137.1 ± 39.3	143.1 ± 51.0
Peak torque 180°/s (N∙m)	106.2 ± 35.9	104.1 ± 32.6	91.1 ± 31.2	91.8 ± 28.3	85.0 ± 37.2	86.3 ± 37.3	103.1 ± 28.2	103.0 ± 35.8
AMT (%)	45.8 ± 7.6	45.7 ± 7.3	48.4 ± 6.8	49.1 ± 6.1	45.1 ± 10.3	44.5 ± 10.6	46.5 ± 9.4	44.0 ± 11.1
Normalized VL MEP amplitude (%)	3.3 ± 2.8	3.3 ± 2.4	2.4 ± 1.4	3.0 ± 2.0	2.4 ± 2.1	2.0 ± 1.4	2.8 ± 0.6	2.8 ± 1.4
VL SP duration (ms)	133.9 ± 16.7	137.1 ± 18.8	126.4 ± 17.3	134.9 ± 18.6	121.0 ± 23.9	128.8 ± 24.2	122.3 ± 11.6	121.0 ± 13.0
Segmental lean mass (kg)	12.2 ± 2.6	11.8 ± 2.2	11.1 ± 1.4	10.6 ± 1.2	10.8 ± 2.1	10.3 ± 2.1	10.5 ± 1.2	10.3 ± 1.2
VL CSA (cm^2^)	25.7 ± 7.9	24.6 ± 8.4	28.1 ± 8.9	27.2 ± 9.1	23.9 ± 6.4	22.1 ± 6.6	23.6 ± 6.4	24.5 ± 6.6
RF CSA (cm^2^)	8.8 ± 2.7	9.0 ± 2.1	6.8 ± 1.5	6.0 ± 1.6	6.6 ± 2.2	6.2 ± 1.5	6.2 ± 0.9	6.1 ± 1.3

**TABLE 2:** Within group changes listed as percentage change (effect size).

Variable	Immobilization	AOMI	NMES	Control
MVC peak torque	−12.7 % (0.820)	−8.9% (0.860)	−8.0% (0.912)	−5.0% (0.582)
VA	−4.0% (0.830)	−4.4% (0.337)	+1.3% (0.231)	+2.3% (0.463)
EET	+0.4% (0.046)	−1.0% (0.086)	+9.2% (0.372)	−3.4% (0.422)
Concentric peak torque – 30°/s	−5.0% (0.947)	+7.2% (1.018)	−1.3% (0.095)	+4.4% (0.181)
Concentric peak torque – 180°/s	−2.1% (0.189)	+0.8% (0.018)	+1.5% (0.161)	−0.1% (0.006)
AMT	−0.2% (0.033)	+1.4% (0.175)	−1.3% (0.244)	−5.4% (0.341)
Normalized VL MEP peak‐to‐peak amplitude	+0.6% (0.010)	+25.4% (0.590)	−16.3% (0.252)	−1.8% (0.044)
VL silent period duration	+2.4% (0.298)	+6.8% (0.782)	+6.4% (0.685)	−1.1% (0.117)
Segmental lean mass	−2.7% (0.433)	−4.9% (1.207)	−4.2% (0.838)	−1.8% (0.850)
CSA—VL	−4.3% (0.724)	−3.1% (0.203)	−7.4% (0.992)	+3.6% (0.345)
CSA—RF	+2.1% (0.169)	−12.2% (0.555)	−6.0% (0.280)	−1.4% (0.073)

*Note*: Effect sizes reported as Cohen's *d*.

### 3.1 Compliance

All 31 participants in the immobilization groups had at least 4 days of accelerometer data for a minimum of 10 h and were considered compliant in accordance with Cook et al. (2006). The average number of days worn was 6.75. There was a significantly higher number of vigorous physical activity minutes (*P* < 0.001) and average steps per day (*P* = 0.008) on the right leg compared to the left (immobilized) leg.

### 3.2 Muscle strength outcomes

#### 3.2.1 MVC peak torque

The covariate PRE MVC torque was significantly related to POST MVC torque (*F* (1,34) = 206.22, *P* < 0.01, ƞ_p_^2^ = 0.858). After controlling for PRE MVC torque, there were no significant differences between groups on POST MVC torque (*F* (3,34) = 0.782, *P* = 0.512). The effect size for the ANCOVA was medium (ƞ_p_^2^ = 0.065). After controlling for PRE MVC torque, the adjusted POST MVC torque marginal means were as follows: Immobilization = 146.97 N∙m, AOMI = 153.59 N∙m, NMES = 154.69 N∙m, Control = 160.62 N∙m.

#### 3.2.2 Concentric isokinetic peak torque

The covariate PRE concentric isokinetic peak torque at 30°/s was significantly related to POST concentric isokinetic peak torque at 30°/s (*F* (1,34) = 132.303, *P *< 0.001, ƞ_p_^2^ = 0.796). After controlling for PRE concentric isokinetic peak torque at 30°/s, there was no significant difference between groups for POST concentric isokinetic peak torque at 30°/s (*F* (3,34) = 1.230, *P *= 0.314). The effect size for the ANCOVA was medium/large (ƞ_p_^2^ = 0.098).

**FIGURE 3:**
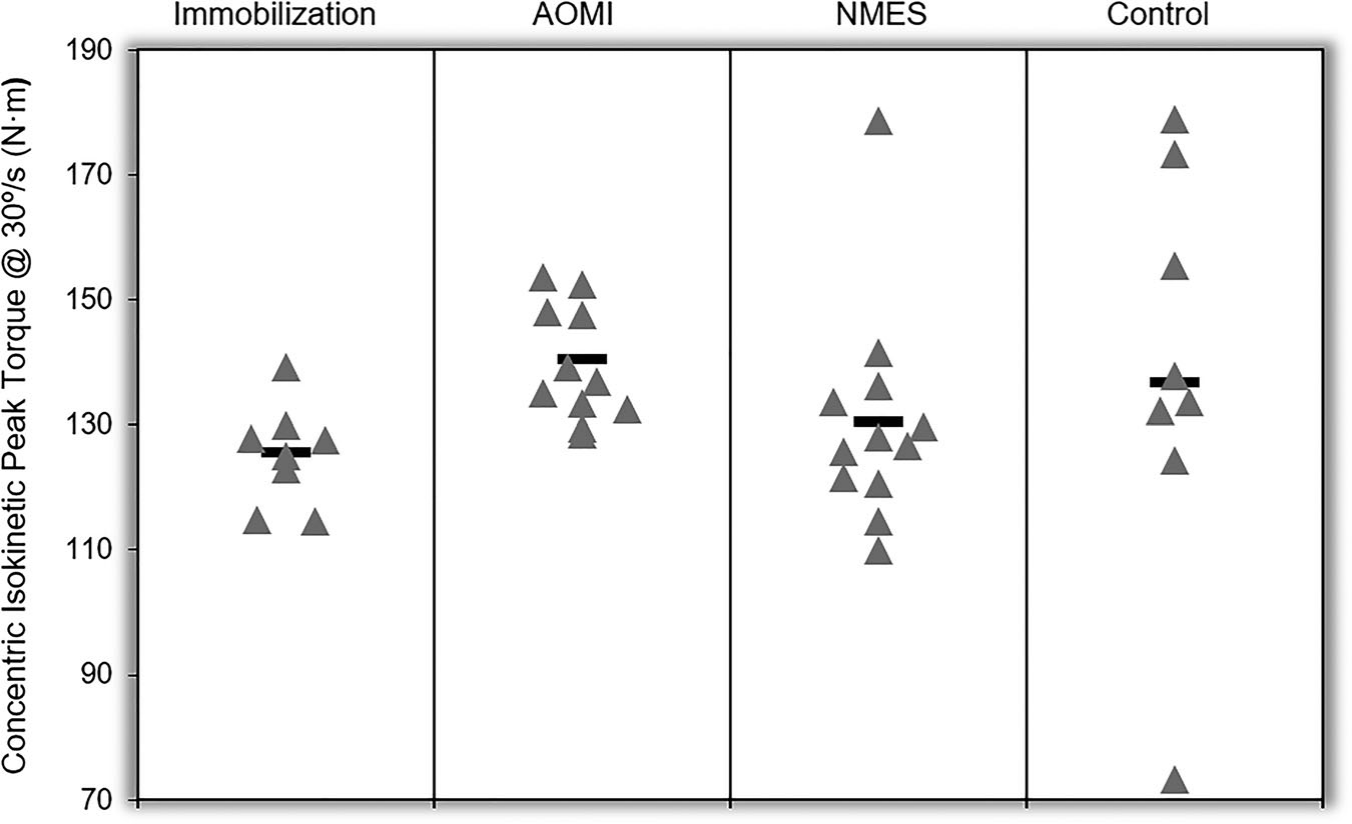
Individual participant ANCOVA‐adjusted POST test values for concentric isokinetic peak torque at 30°/s (N∙m). Black bars represent the mean of each group.

After controlling for PRE concentric isokinetic peak torque at 30°/s, the adjusted marginal means were as follows: Immobilization = 125.77 N∙m, AOMI = 140.75 N∙m, NMES = 130.63 N∙m, Control = 138.71 N∙m. While the results of the ANCOVA indicated no significant difference between groups, based on the ANCOVA‐adjusted marginal means, AOMI peak torque was 11.9% higher than the immobilization group, 7.7% higher than the NMES group and 1.5% higher than the control group (Figure 3).

The covariate PRE concentric isokinetic peak torque at 180°/s was significantly related to POST concentric isokinetic peak torque at 180°/s (*F* (1,43) = 194.145, *P* < .001, ƞ_p_^2^ = 0.851). After controlling for PRE concentric isokinetic peak torque at 180°/s, there was no significant difference between groups for POST concentric isokinetic peak torque at 180°/s (*F* (3,34) = 0.040, *P* = 0.989). The effect size for the ANCOVA was small (ƞ_p_^2^ = 0.004). After controlling for PRE concentric isokinetic peak torque at 180°/s , the adjusted marginal means were as follows: Immobilization = 93.84 N∙m, AOMI = 95.55 N∙m, NMES = 95.66 N∙m, Control = 95.64 N∙m.

#### 3.2.3 Voluntary activation

The covariate PRE VA was significantly related to POST VA (*F* (1,34) = 16.686, *P *< 0.01, ƞ_p_^2^ = 0.329). After controlling for PRE VA, there was no significant difference between groups for POST VA (*F* (3,34) = 2.249, *P *= 0.100). The effect size for the ANCOVA was large (ƞ_p_^2^ = 0.166). After controlling for PRE VA, the adjusted marginal means were as follows: Immobilization = 90.01%, AOMI = 87.62%, NMES = 93.93%, Control = 95.35%.

#### 3.2.4 Electrically evoked torque

The covariate PRE EET was significantly related to POST EET (*F* (1,34) = 162.221, *P *< 0.001, ƞ_p_^2^ = 0.827). After controlling for PRE EET, there was no significant difference between groups for POST EET (*F* (3,34) = 1.014, *P *= 0.399). The effect size for the ANCOVA was medium (ƞ_p_^2^ = 0.082). After controlling for PRE EET, the adjusted marginal means were as follows: Immobilization = 71.52 N∙m, AOMI = 70.27 N∙m, NMES = 76.85 N∙m, Control = 68.96 N∙m.

### 3.3 Neural outcomes

#### 3.3.1 Active motor threshold

The covariate PRE AMT was significantly related to POST AMT (*F* (1,34) = 115.673, *P* < .001, ƞ_p_^2^ = 0.773). After controlling for PRE AMT, there was no significant difference between groups for POST AMT (*F* (3,34) = 0.899, *P* = 0.452). The effect size for the ANCOVA was medium (ƞ_p_^2 ^= 0.074). After controlling for PRE AMT, the adjusted marginal means were as follows: Immobilization = 46.22% stimulator output, AOMI = 47.26% stimulator output, NMES = 45.69% stimulator output, Control = 43.90% stimulator output.

#### 3.3.2 Normalized motor evoked potential amplitude

The covariate normalized PRE VL MEP amplitude was significantly related to normalized POST VL MEP amplitude (*F* (1,34) = 25.714, *P *< 0.001, ƞ_p_^2^ = 0.431). After controlling for normalized PRE VL MEP amplitude, there was no significant difference between groups for normalized POST VL MEP amplitude (*F* (3,34) = 1.038, *P* = 0.388). The effect size for the ANCOVA was medium (ƞ_p_^2^ = 0.084). After controlling for normalized PRE VL MEP amplitude, the adjusted marginal means were as follows: Immobilization = 2.9%, AOMI = 3.2%, NMES = 2.2%, Control 2.7%. While the results of the ANCOVA indicated no significant difference between groups, based on the ANCOVA‐adjusted marginal means, normalized MEP amplitude in the AOMI group was 10.3% higher than that of the immobilization group, 45.5% higher than that of the NMES group and 18.5% higher than that of the control group.

**FIGURE 4:**
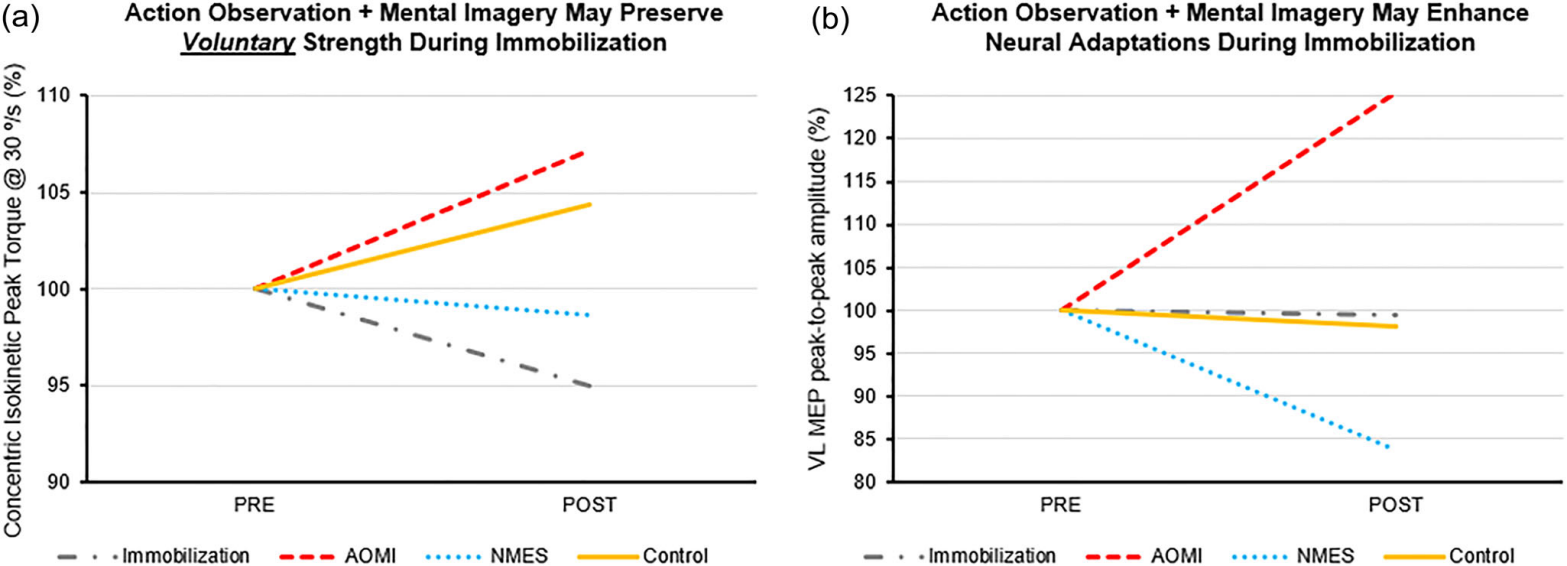
Comparison of within group PRE to POST percent changes for (a) concentric isokinetic peak torque at 30°/s and (b) normalized VL MEP peak‐to‐peak amplitude.

A comparison of voluntary strength and MEP amplitude can be seen in Figure 4.

#### 3.3.3 Silent period duration

The covariate PRE VL silent period duration was significantly related to POST VL silent period duration (*F* (1,34) = 79.146, *P* < .001, ƞ_p_^2^ = 0.700). After controlling for PRE VL silent period duration, there was no significant difference between groups for POST VL silent period duration (*F* (3,34) = 1.548, *P* = 0.220). The effect size for the ANCOVA was large (ƞ_p_^2^ = 0.120). After controlling for PRE VL silent period duration, the adjusted marginal means were as follows: Immobilization = 129.76 ms, AOMI = 134.26 ms, NMES = 132.83 ms, Control = 123.94 ms.

### 3.4 Muscle mass and morphology outcomes

#### 3.4.1 Segmental lean mass

The covariate PRE segmental lean mass was significantly related to POST segmental lean mass (*F* (1,34) = 437.260, *P* < .001, ƞ_p_^2^ = 0.928). After controlling for PRE segmental lean mass, there was no significant difference between groups on POST segmental lean mass (*F* (3,34) = 1.112, *P* = 0.358). The effect size for the ANCOVA was medium (ƞ_p_^2 ^= 0.089). After controlling for PRE segmental lean mass, the adjusted POST segmental lean mass marginal means were as follows: Immobilization = 10.92 kg, AOMI = 10.58 kg, NMES = 10.63 kg, Control = 10.85 kg.

#### 3.4.2 Muscle CSA

The covariate PRE VL CSA was significantly related to POST VL CSA (*F* (1,34) = 237.889, *P* < .001, ƞ_p_^2^ = 0.875). After controlling for PRE VL CSA, there was no significant difference between groups for POST VL CSA (*F* (3,34) = 1.441, *P* = 0.248). The effect size for the ANCOVA was large (ƞ_p_^2 ^= 0.113). After controlling for PRE VL CSA, the adjusted marginal means were as follows: Immobilization = 24.22 cm^2^, AOMI = 24.53 cm^2^, NMES = 23.52 cm^2^, Control = 26.12 cm^2^.

The covariate PRE RF CSA was significantly related to POST RF CSA (*F* (1,34) = 43.320, *P* < 0.01, ƞ_p_^2^ = 0.560). After controlling for PRE RF CSA, there was a significant difference between groups for POST RF CSA (*F* (3,34) = 3.837, *P* = 0.018). The effect size for the ANCOVA was large (ƞ_p_^2 ^= 0.253). Results of the Bonferroni *post hoc* testing indicated that POST RF CSA was significantly smaller (*P *= 0.014) in the AOMI group (6.16 cm^2^) than the immobilization‐only group (7.94 cm^2^). After controlling for PRE RF CSA, the adjusted marginal means were as follows: Immobilization = 7.94 cm^2^, AOMI = 6.16 cm^2^, NMES = 6.52 cm^2^, Control = 6.68 cm^2^.

### 3.5 Nutritional intake

The results of the one‐way ANOVA tests revealed no significant difference between groups for calorie (*F* (3,35) = 0.528, *P* = 0.666, ƞ_p_^2^ = 0.043) or protein intake (*F* (3,35) = 2.012, *P* = 0.130). However, the effect size for protein was large (ƞ_p_^2^ = 0.147). The mean ± SD (range) calorie intake per group was as follows: Immobilization = 1887.96 ± 415.06 (1251.00–2492.67) calories, AOMI = 1640.33 ± 440.72 (854.67–2322.33) calories, NMES = 1633.08 ± 742.42 (737.33–3131.00) calories, Control = 1808.25 ± 400.35 (1182.33–2283.33) calories. The mean ± SD (range) protein intake per group was as follows: Immobilization = 104.11 ± 31.04 (70.67–160.00) g, AOMI = 73.03 ± 24.02 (33.67–97.33) g, NMES = 77.36 ± 29.09 g (31.33–126.33), Control = 90.79 ± 39.22 (50.33–155.67) g.

## 4 DISCUSSION

Previous studies have shown dissociations and divergent timelines between muscle weakness and atrophy during disuse, highlighting the need for therapeutic interventions that mechanistically and uniquely target the nervous system versus skeletal muscle. We sought to determine if disuse‐induced deficits in muscle strength, mass and neuromuscular function during 1 week of knee joint immobilization were mitigated with the addition of AOMI or NMES interventions. All immobilization groups generally experienced strength loss, muscular atrophy and neuromuscular deficits. While the results of the ANCOVA tests were not statistically significant, an overreliance on solely null hypothesis significance testing does not provide information on magnitude of effect as it may relate to practical or clinical implications (Wasserstein et al., 2019; Williams et al., 2023). Based on observed effect sizes and ANCOVA‐adjusted means, it appears that AOMI preserved voluntary strength (particularly at 30°/s), which may have been modulated by increased corticospinal excitability, and aligns with our hypothesis. However, NMES did not preserve muscle mass, which contradicts our hypothesis. Based on these results, we believe that AOMI shows promise for therapeutically targeting neural plasticity and preventing voluntary strength loss during immobilization.

In examining the current intervention, it is important to begin with whether it was an effective model for inducing loss of muscle strength and mass. Considering the immobilization‐only group in the current study, it is evident that strength was negatively impacted by 1 week of immobilization, as MVC peak torque decreased ∼12%. Muscle mass was less affected, with the immobilization group experiencing a 4.3% decline in VL CSA and no measurable decrease in RF CSA. This is typical of the immobilization literature, as strength has been observed to decrease more rapidly than muscle mass (Dirks et al., 2014; S. W. Jones et al., 2004; Thom et al., 2001; Wall et al., 2014; White et al., 1984), likely due to factors contributing to neuromuscular deconditioning (Clark & Manini, 2008), such as decrements in VA and corticospinal responses (Clark et al., 2014).

### 4.1 Action observation/mental imagery

Based on the observed effect sizes and within group changes, the results of this intervention indicate that AOMI shows promise in preserving voluntary strength, which may be modulated by increases in corticospinal excitability. In addition to demonstrating a medium/large effect, the ANCOVA‐adjusted POST test value for AOMI concentric isokinetic peak torque at 30°/s was 11.9% higher than that of the immobilization‐only group. Given the challenge of recovering from periods of immobilization, these differences in outcomes may be clinically meaningful.

There are also several interesting observations when considering the PRE to POST changes within the AOMI group alone. Of particular interest is that the AOMI intervention resulted in an increase in isokinetic peak torque at 30°/s by 7.2% (Table 2). Given that the AO video consisted entirely of heavy knee extensions viewed from a variety of perspectives, this finding likely has implications for specificity of AO interventions via activation of the mirror neuron system. In support of this, successful AO interventions have typically employed movements that reflect daily actions or are similar to the actions in which an effect is desired (Bellelli et al., 2010; Buccino, 2014; Maeda et al., 2002; Naish et al., 2014). While the intent of the videos was to show the VL and RF muscles actively working through muscle contractions, it is possible that the primary outcome measure for strength (i.e., MVC peak torque) would have been better preserved if the videos had been isometric in nature. However, as this is difficult to display, it may be more practical to assess dynamic strength outcomes that mirror AOMI interventions PRE and POST immobilization.

In support of our hypothesis that AOMI interventions may preserve strength via neural factors, the increase in isokinetic concentric peak torque at 30°/s for the AOMI group is likely related to the neuromuscular system, as VL and RF CSA decreased 3.1% and 12.2%, respectively. Further, RF CSA was significantly less in the AOMI group than in the immobilization‐only group. While there were minimal changes in VA (−4.4%) and silent period duration (+6.8%), changes in corticospinal excitability were apparent. From PRE to POST, the AOMI intervention resulted in a 25.4% increase in normalized VL MEP amplitude (Table 2), which may indicate AOMI shows promise in strength maintenance via positive corticospinal adaptations.

There are several methodological considerations which may have strengthened our findings and should be considered in future studies. Effective AO and MI interventions have often occurred within a laboratory setting (Clark et al., 2014; Ranganathan et al., 2004; Yue & Cole, 1992), which was not the case in the present study. While participants were instructed to perform AOMI sessions in a quiet area on a full computer screen (rather than on their phone or tablet), it is possible that they did not comply with this request. Unfortunately, compliance officers reported that on one documented occasion, a participant fell asleep during the AOMI intervention. Another participant remarked that they were bored during the AOMI sessions. While the goal of the sessions was not to be entertaining, it is possible that a lack of focus or simply ‘going through the motions’ may have prevented more robust preservation of strength and neuromuscular function. In support of this, there is evidence in the literature that both strength and corticospinal excitability outcomes during AOMI are dependent on the level of observed/imagined intensity (Harmon et al., 2022; Helm et al., 2015; Mizuguchi et al., 2013; Ranganathan et al., 2004). While positive changes may occur with simple exposure to AOMI interventions, effort and intention would likely increase the effects.

There has also been recent attention on utilizing the PETTLEP model for successful AOMI interventions (Wright et al., 2021). The PETTLEP model outlines seven factors related to MI: Physical, Environment, Task, Timing, Learning, Emotion, Perspective. These factors have been demonstrated as beneficial when utilizing MI for improved sports performance (Smith et al., 2007) and therefore are likely an important consideration in combined AOMI interventions. While we were able to control for certain factors (e.g., Task and Perspective), we were unable to control for others (e.g., Environment and Emotion). While we did not track previous training experience, many of the participants remarked on having never performed knee extensions previously. It is possible that those participants who had previous experience with the contents of the AOMI interventions (e.g., knee extensions, a gym setting) may have been better able to perform kinaesthetic mental imagery while performing the AOMI sessions. Had we targeted individuals with previous resistance training experience, it is possible that the outcomes of AOMI would have been improved to a greater extent through participant familiarity with the AOMI subject matter.

### 4.2 Neuromuscular electrical stimulation

Contrary to our hypothesis, NMES did not exert a preservatory effect on muscle mass. While every effort was made to replicate the intervention by Dirks et al. (2014) insofar as the NMES training protocol (e.g., electrode placement, stimulation ramp time, contraction time, relaxation time, frequency, and pulse length), we were unable to replicate their findings of maintaining muscle mass. Rather, the NMES group experienced 7.4% and 6.0% decreases in VL and RF CSA, respectively (Table 2). This may be due to several methodological differences between the two studies. Dirks et al. (2014) measured muscle size with CT scans whereas the current study utilized B‐mode ultrasound. While there are inconsistent reports in the literature as to whether CT is more accurate than B‐mode ultrasound (Smith‐Bindman et al., 2014; Suri et al., 1999; van Randen et al., 2011), utilizing two different techniques for measuring muscle mass may have resulted in differing conclusions. Furthermore, differences in the immobilization protocol may have resulted in inconsistent outcomes, as Dirks et al. (2014) immobilized participants in a full leg cast at 30° knee flexion, compared to the use of a removable leg brace set at 90° knee flexion in the present study, which may have allowed for more inadvertent movement at the hip.

Additionally, protein intake was somewhat low in the present study, with some individuals eating as little as 10–20 g of protein daily. While protein consumption does not appear to mitigate disuse‐induced deficits or changes in protein synthesis/breakdown during immobilization (Kilroe et al., 2021), it is possible that the combination of low protein intake, immobilization, and repetitive muscle contractions via twice daily NMES resulted in a particularly catabolic environment, with multiple factors contributing to muscle protein breakdown, combined with decreased protein synthesis observed during immobilization (Howard et al., 2020; Kilroe et al., 2020, 2021; Tesch et al., 2008; Wall & van Loon, 2013) and subpar nutritional intake. Increased MAFbx and MuRF1 mRNA expression have been observed during immobilization, both of which play key roles in the ubiquitin‐proteasome pathway thought to regulate muscle protein breakdown (Dirks et al., 2014). While NMES has been observed to prevent upregulated expression of these ligases (Dirks et al., 2014), it is possible that a lack of intensity combined with poor nutritional intake in the present study was not able to overcome the immobilization‐induced increase in muscle protein breakdown and decrease in rates of protein synthesis. However, given that muscle biopsies were not obtained herein, this explanation is speculative.

Despite the lack of findings between groups, it may be of interest to note that in examining PRE to POST changes within the NMES group, EET capacity was improved by 9.2% (Cohen's *d* = 0.372), whereas the immobilization‐only group showed minimal differences from PRE (+0.4%, Cohen's *d* = 0.046) (Table 2). This may be meaningful, as both muscle mass and contractile properties have been postulated to be indirectly measurable through resting EET (Behrens et al., 2016). Although NMES did not result in preservation of muscle mass herein, the 9.2% increase in EET in the NMES group is notable, as previous evidence indicates that EET may be considered a metric for intrinsic muscle force generating capacity (Palmieri‐Smith et al., 2021). EET provides insight into the ability of the muscle contractile tissue to produce force and is considered a surrogate measure of skeletal muscle mechanical function (Brass et al., 1996; Krishnan & Williams, 2009). While a great deal of strength production is neural, EET bypasses neural influence, providing insight into the involuntary force capacity of a muscle (Krishnan & Williams, 2009). Further, EET may provide insight into other peripheral adaptations which were not measured in the present study, such as changes in muscle morphology and tendon architecture (Davies et al., 1985; Narici et al., 2003).

There is some speculation that NMES may exert preservatory effects on muscle mass via recruitment of primarily type II fibres (Dirks et al., 2014), those classically considered responsible for strength production. While we did not obtain muscle biopsies or measure fibre size, it is possible that greater EET in the NMES group was related to routine activation of type II fibres via twice daily stimulation sessions. While the precise mechanism is uncertain, it is possible that the regular contractions due to NMES helped to maintain involuntary performance, despite no maintenance of voluntary strength or muscle mass.

### 4.3 Clinical relevance

There are several findings herein which may be clinically important when considering the between‐group outcomes. Primarily, dynamic strength (as measured by concentric isokinetic peak torque at 30°/s) was best maintained in the AOMI group, resulting in peak torque 11.9% higher than the immobilization group based on the ANCOVA‐adjusted marginal means. In support of the clinical importance of these findings, a recent study by Kirn et al. (2016) noted an improvement in leg extensor power of 9–10% to be considered clinically meaningful. Given that recovery from periods of prescribed immobilization can be prolonged and difficult, an intervention resulting in greater strength outcome could potentially lessen the duration of rehabilitation needed. As such, the observation of a greater level of strength after immobilization should not be discounted, as a difference as seemingly little as 10% has the potential to substantially improve rehabilitation timelines.

### 4.4 Strengths of our approach

Given the challenging nature of large‐scale immobilization studies, the sample size herein is noteworthy. Several excellent immobilization studies have sample sizes of ∼5–15 participants (Clark et al., 2008, 2006, 2014, 2010; Cook et al., 2014; MacLennan et al., 2021, 2020). Overall compliance with the immobilization protocol was also excellent, with 100% compliance for all 31 immobilized participants. This was likely aided by the compliance officers, who ensured that participants had regular contact with the research team, allowing problems to be addressed quickly if necessary. This study also implemented a robust assessment protocol, utilizing both muscular and neural assessments, allowing for a more complete picture of disuse‐induced adaptations. Finally, the inclusion of a true control group is an important addition, allowing for a more thorough examination of changes to the intervention (Atkinson & Batterham, 2015).

### 4.5 Limitations

This study has limitations worthy of discussion. While overall compliance with the immobilization protocol was excellent, it is possible that compliance with the AOMI and NMES interventions was subpar. While we did everything possible to ensure the interventions were being performed (e.g., thorough FAM, daily check‐ins from compliance officers), participants may have failed to perform their AOMI or NMES sessions consistently or with appropriate intensity for greater differences in outcome measures to be captured. Future studies may benefit from efforts to ensure attentional focus during AOMI and proper progression of intensity during NMES, possibly during laboratory visits or virtual sessions with a member of the research team monitoring the intervention protocol.

While the inclusion of a true control group was a strength of the study, the control group demonstrated several undesirable changes from PRE to POST. MVC peak torque decreased by 5% in the control group, while VL CSA increased 3.6%. Although we are uncertain as to the reasons for the negative changes, these fluctuations likely confounded the overall interpretation of results.

BIS, an important assessment in this study, is well known to be heavily influenced by hydration status (El‐Kateb & Davenport, 2016; Francisco et al., 2021; Schotman et al., 2021). However, we were unable to track hydration status, and did not account for phase of the menstrual cycle, which can largely influence fluid retention (Stachenfeld, 2008). Therefore, hydration status may have impacted segmental lean mass results.

In retrospect, it appears that an additional limitation is the lack of specificity between the AOMI intervention and the isometric MVCs, considered the primary outcome variable to assess strength. Given the increase in concentric isokinetic peak torque at 30°/s in the AOMI group, there appears to be a significant specificity component to AOMI interventions. The results herein may have been even more pronounced had our primary outcome variable more closely matched the AOMI intervention. Similarly, we did not track training experience, which may be beneficial in best utilizing the PETTLEP model of AOMI. The more participants can closely relate to the AOMI interventions, the better outcomes may be. Had participants been more familiar with the feeling of performing knee extension exercises, they may have been better able to engage in kinaesthetic MI.

Finally, the physiology behind immobilization without trauma likely differs from immobilization with associated trauma. After trauma or injury, there is an innate physiological response that results in pain, inflammation and swelling of the affected area (Cooke, 2019), which further results in a decline in range of motion, neuromuscular signalling, and muscular strength capacity (Howard et al., 2020). Conversely, in immobilization models where otherwise healthy limbs undergo a period of disuse, the physiological responses associated with trauma are not present. Therefore, it is possible that the results obtained herein may differ in patients recovering from injury or surgery.

### 4.6 Conclusions

In conclusion, during 1 week of unilateral knee joint immobilization, AOMI may show promise as a means to preserve voluntary strength. Given the differences between and within groups in muscle strength outcomes, AOMI may enhance rehabilitation protocols by preserving strength via positive neural adaptations. Specificity of observed/imagined actions appears to play a significant role in successful AOMI interventions, and future investigations should examine AOMI interventions that closely mirror the chosen strength outcome measures.

## AUTHOR CONTRIBUTIONS

Conceptualization: Kylie K. Harmon, Ryan M. Girts and Matt S. Stock. Methodology: Kylie K. Harmon, Ryan M. Girts, Joshua C. Carr, Michael D. Roberts, Debbie L. Hahs‐Vaughn, David H. Fukuda, Jeffrey R. Stout and Matt S. Stock. Software: Matt S. Stock and Jeanette Garcia. Validation: Kylie K. Harmon, Ryan M. Girts, Gabriela Rodriguez, Jeanette Garcia and Matt S. Stock. Formal analysis: Kylie K. Harmon, Ryan M. Girts, Debbie L. Hahs‐Vaughn, Gabriela Rodriguez and Matt S. Stock. Investigation: Kylie K. Harmon, Ryan M. Girts, Jonathan P. Beausejour and Jason I. Pagan. Resources: Jeanette Garcia and Matt S. Stock. Data curation: Kylie K. Harmon, Ryan M. Girts, Gabriela Rodriguez and Matt S. Stock. Writing—original draft preparation: Kylie K. Harmon. Writing—review and editing: Ryan M. Girts, Gabriela Rodriguez, Jonathan P. Beausejour, Jason I. Pagan, Joshua C. Carr, Michael D. Roberts, Debbie L. Hahs‐Vaughn, Jeanette Garcia, David H. Fukuda, Jeffrey R. Stout and Matt S. Stock. Visualization: Kylie K. Harmon and Matt S. Stock. Supervision: Kylie K. Harmon, Ryan M. Girts and Matt S. Stock. Project administration: Kylie K. Harmon and Ryan M. Girts. Funding acquisition: Ryan M. Girts. All authors have read and approved the final version of this manuscript and agree to be accountable for all aspects of the work in ensuring that questions related to the accuracy or integrity of any part of the work are appropriately investigated and resolved. All persons designated as authors qualify for authorship, and all those who qualify for authorship are listed.

## CONFLICT OF INTEREST

The authors declare no conflicts of interest.

## Supporting information

Table S1.

## Data Availability

Queries related to these data can be addressed by K.K.H. kkharmon@syr.edu. Supplementary information can be found in Table S1 and raw data will be provided upon reasonable request.
